# Impact of adolescent social Isolation on increased risk for alcohol intake and aversion-resistant drinking in males and females

**DOI:** 10.21203/rs.3.rs-9859563/v1

**Published:** 2026-06-25

**Authors:** Andrew R. Burke, Morgan Sullivan, Catrina Pereda, Gabby Reser, Olivia McIntosh, Stephanie Arenas, Nolen Cunningham, Frederic W. Hopf, Jodi L. Lukkes

**Affiliations:** Indiana University School of Medicine; Indiana University School of Medicine; Indiana University School of Medicine; Indiana University School of Medicine; Indiana University School of Medicine; Indiana University School of Medicine; Indiana University School of Medicine; Indiana University School of Medicine; Indiana University School of Medicine

**Keywords:** adolescence, social isolation, alcohol, aversion-resistant drinking

## Abstract

**Rationale:**

Adverse experiences during adolescence, especially social isolation, increases risk for alcohol use disorders (AUDs). Previous rodent studies of adolescent social isolation (ASI) encompassed all stages of adolescence and/or maintained isolation into adulthood, limiting identification of vulnerable stages of adolescence. Utilizing males only, or combining the sexes, also may have contributed to contradictory findings.

**Objectives:**

Investigate social isolation restricted to discrete adolescent stages in combination with re-socialization and then persistent alcohol drinking. Determine whether adolescent isolation during specific developmental time periods, with or without subsequent re-socialization, leads to increased alcohol drinking in males or females, examined separately.

**Methods:**

Male and female Wistar rats underwent one of six housing conditions: 1) isolation from postnatal day (P)21–42 followed by re-socialization (ASIR-Early), 2) isolation from P42–63 followed by re-socialization (ASIR-Late), or 3) continuous isolation from P21 onward (ASI), or 4–6) one of three appropriate control treatments. Alcohol-related behaviors were assessed in adulthood using intermittent-access two-bottle choice drinking (IAP), limited daily access (LDA-20) procedures, quinine-adulterated aversion-resistant drinking (ARD), and alcohol re-exposure following prolonged abstinence.

**Results:**

In general, ASIR-Early and ASI elevated alcohol drinking in males and females, when compared to contemporary controls, during IAP, LDA-20, ARD, and after prolonged withdrawal, while ASIR-Late had largely similar effects as control conditions. However, for females, only ASI led to greater ARD at the highest quinine dose and also increased alcohol drinking after prolonged withdrawal. In contrast, for males, ASIR-Early and ASI had largely similar enhancement of alcohol drinking.

**Conclusions:**

Under these conditions, isolation housing that encompassed the early adolescent period produced enduring increases in alcohol-related behaviors, with re-socialization perhaps more protective in females against ARD and elevated intake after abstinence. These results inform future studies that will investigate neural mechanisms underlying ASI-induced enhancement of alcohol drinking.

## Introduction

Alcohol misuse imposes a substantial burden on society, costing hundreds of billions of dollars annually and negatively impacting hundreds of millions of individuals worldwide (NIAAA.NIH.org). Identifying factors that increase risk for alcohol use disorder (AUD) and developing effective preventative strategies could save billions of dollars and millions of lives. We know that adolescence is a period during which negative experiences can have profound influences on final maturation of the brain and thus impact how vulnerable the person is that emerges in early adulthood to developing AUD.

The Adverse Childhood Experiences (ACE) study—the largest investigation of negative early-life experiences to date (Felitti et al. 1998)—demonstrated that higher ACE scores are strongly associated with increased risk for AUD (Dube et al. 2002; Dube et al. 2006; Loudermilk et al. 2018; Mersky et al. 2013). The likelihood of developing a substance use disorder, such as AUD, increased as ACE indices increase (LeTendre and Reed 2017). Importantly, specific social stressors during development, including adolescent social victimization and early-life loneliness, are also linked to increased alcohol use and alcohol-related problems (Berberian et al. 2022; Topper et al. 2011). Disengagement from one’s social network is associated with greater risk for substance misuse (Copeland et al. 2018), especially in females (below). However, much remains unclear about whether isolation during specific periods of adolescence is more susceptible to conferring long-term vulnerability to AUD later in adulthood, and addressing such concerns could provide a valuable framework for future studies to identify critical brain changes related to later pathological behavior.

To address this gap, reliable animal models are essential for isolating, manipulating, and characterizing the behavioral and neural consequences of adolescent adversity. Rats provide a valuable model due to their highly social nature, particularly during adolescence, when peer-directed interactions carry strong motivational significance and are critical for the development of social competence (Douglas et al. 2004; Vanderschuren et al. 1997). A substantial body of research suggests that adverse social experiences during rat adolescence, including adolescent social isolation (ASI), produces behavioral phenotypes that can be considered to reflect mental illnesses and addiction (Burke et al. 2017; Butler et al. 2014a; Butler et al. 2016; Chandler et al. 2022; Orben et al. 2020; Skelly et al. 2015). For example, social isolation during early adolescence (postnatal day (P) 21 to P42) followed by re-socialization (termed ASIR-Early in the present study) reliably exacerbates negative behavioral outcomes during adulthood in our previous rat studies (i.e., Burke et al. 2017; Lukkes et al. 2012b; Lukkes et al. 2009b; Lukkes et al. 2009d) and studies by others (i.e., Cuesta et al. 2020; Makinodan et al. 2012; Whitaker et al. 2013; Zhang et al. 2019). These negative behavioral phenotypes, which include anxiety- and depression-like behaviors, are most likely the result of neurobehavioral alterations that occur during this sensitive and discrete developmental period.

Importantly, clinical evidence indicates that women are more susceptible than men to early-life adversity, which can lead to greater cue reactivity to alcohol in experimental settings (Hartwell and Ray 2013) and increased vulnerability for alcohol relapse (Heffner et al. 2011; Hyman et al. 2008; Kennedy et al. 2013) when compared to men. Adolescent girls also exhibit a hypersensitivity to peer influence (O'Brien and Bierman 1988; Sebastian et al. 2010a; Sebastian et al. 2010b), and adolescent females may be particularly vulnerable to adverse alcohol-related outcomes associated with loneliness (McKay et al. 2017). However, prior rodent studies have reported inconsistent effects of adolescent isolation on anxiety-like behavior and alcohol intake, and, in particular, sex differences in preclinical models of ASI and alcohol use remain understudied (McCarthy et al. 2012). Some early studies failed to observe increased alcohol consumption following adolescent isolation (Fahlke et al. 1997; Thorsell et al. 2005), while others report increased drinking (Deatherage 1972; Hall et al. 1998; Juarez and Vazquez-Cortes 2003). For example, in male rats, ASI increased anxiety-like behavior and alcohol intake in adulthood versus group-reared rats (Chappell et al. 2013; McCool and Chappell 2009; Skelly et al. 2015), similar to male rats isolated only for adolescence (Lesscher et al. 2015). However, ASI sex effects may also vary between rats (Butler et al. 2014b) and mice (Rivera-Irizarry et al. 2020).

One important consideration is that sex differences in the impact of adolescent isolation may be confounded by the stress of adult solitary housing, which may be significant for females than males (Brown and Grunberg 1995). For example, isolated adult females drink more in relapse compared to group-housed females (Moench and Logrip 2020). Also, many previous ASI studies maintain animals in isolation from weaning through adulthood and assess behavior during ongoing isolation and compare findings to rats who begin isolation as adults. Thus, continuous isolation makes it difficult to disentangle adolescence-specific effects from adulthood isolation, and our paradigm is designed to address and overcome these limitations. Indeed, only a few studies have compared short periods of isolation (Lesscher et al. 2015).

Thus, in the present study, we sought to restrict isolation to early- or late-adolescence, followed by re-socialization, to narrow down when behavioral alterations occur in a more precise manner, and to compare isolation to appropriate controls (i.e., re-socialized controls) within the same study. Thus, our ASIR-Early and ASIR-Late paradigms refine standard adolescent isolation models by restricting social isolation to discrete periods spanning early- to mid-adolescence. We discovered that both males and females reared in isolation during early adolescence, or throughout all of adolescence, exhibited elevated alcohol drinking in some form or another. Females isolated from weaning onward exhibited the most aversion-resistant alcohol drinking, failing to reduce drinking even at the highest dose of quinine.

## Methods

### Animals

144 Male and female Wistar rats (n = 6–18/group; Envigo) arrived at P21 and were either housed individually or in groups of three per cage with lights off at 10 am and on at 10 pm with *ad libitum* food and water. All experimental procedures were conducted in accordance with the Guide for Care and Use of Laboratory Animals provided by the National Institutes of Health and approved by the Institutional Animal Care and Use Committee of Indiana University. All efforts were undertaken to reduce the number of animals needed and to minimize pain and suffering.

### Social isolation Paradigm

**Figure 1:**
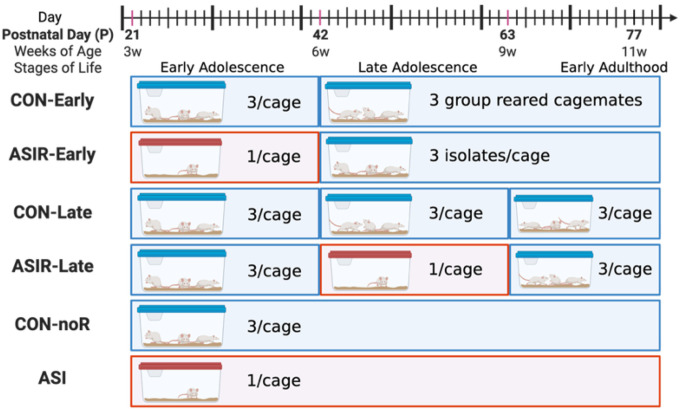
Schematic of housing conditions for adolescent social isolation and re-socialization during early adolescence (ASIR-Early) and controls (CON-Early) or ASIR during late adolescence (ASIR-Late) and controls (CON-Late); or continuous adolescent social isolation (ASI) and controls with no re-socialization (CON-noR).

Based on our past, rats were split into three separate social isolation protocols (Fig. 1). 1) As previously investigated (Lukkes et al. 2009a; Lukkes et al. 2012a; Lukkes et al. 2012b; Lukkes et al. 2009b; Lukkes et al. 2009c; Lukkes et al. 2009d), ASIR-Early rats were socially isolated for three weeks from P21 to P42 from early to mid-adolescence. Animals were then re-socialized at P42 until behavioral testing around P80. Re-socialization consisted of placing isolates with isolates (3/cage). The controls were group-housed with 3/cage (CON-Early) from P21 onward and then re-socialized with a new set of group-reared rats at P42. The re-socialization period ensured that any observed changes were due to social isolation during a discrete period of development. 2) A second group of rats were group-housed (3/cage) for three weeks from P21-P42. On P42, animals were then split into group-reared (CON-Late; 3/cage) or individually-housed (ASIR-Late; 1/cage) for three weeks from P42 to P63 from late adolescence to emerging adulthood, similar to a previous study (Whitaker et al. 2013). Animals were then re-socialized (3/cage) starting from P63. If the rats grew too big (> about 400 grams) they were changed to 2 per cage. Like group 1, socially isolated were re-socialized with 3 previously isolated rats, and group-housed re-housed with 3 new group-housed rats. 3) The third housing condition mimicked conventional isolation paradigms (Hall 1998). Subjects were isolated starting at P21 and remained in isolation throughout the entire adolescent period in a state of social deprivation (ASI) or they were housed in groups with no re-socialization (CON-noR). Finally, all rats were then single housed at 80 days old, when behavioral testing began, since our alcohol drinking paradigms required only one rat per cage.

#### 2-Bottle Choice Intermittent Access Protocol (IAP) and limited daily access (LDA)

Starting on P80 in adulthood, all rats were individually housed into a cage with two holes that allowed for custom-created two-bottle holders to be attached to the front of their home cage to accommodate angled sippers. Rats were ~250–450 g at the time of experimental studies. All consumption occurred in the home cage, with the spout of drinking bottles inserted through holes in the front of the home cage ~7 cm above the cage floor. Rats were provided with 2 bottles on the home cage, one containing 20% alcohol (v/v in water) and one containing water (Moench and Logrip 2020). Alcohol and water bottles were available on alternate days for 24 h (e.g. Monday, Wednesday, Friday (Simms et al. 2008; Wise 1973). Fluid consumption was determined by changes in bottle weight, measured before and after 24-h access across the experiment for 13 weeks. After four weeks of IAP, we also then measured alcohol intake for the first hour, when the drive to consume is highest (Carnicella et al. 2014). The alcohol and water bottle positions were alternated across days to prevent a position bias. Several studies have shown that Wistar and other outbred rat strains need at least 3 months of IAP to develop aversion-resistant drinking (Hopf et al. 2010; Seif et al. 2013; Seif et al. 2015; Spoelder et al. 2015; Spoelder et al. 2017). Thus, we allowed rats ~ 3 months of IAP, at which point rats were shifted to limited daily access two-bottle choice (LDA), with 20 min access to 20% alcohol or water Monday through Friday (Hopf et al. 2010; Seif et al. 2013).

### Aversion-resistant drinking (ARD)

After at least two to three weeks of LDA, rats were tested for willingness to drink alcohol despite addition of the aversive tastant quinine. ARD testing occurred 1–2 times/week, with at least one unadulterated alcohol drinking session between ARD tests, to prevent lasting decrements in alcohol intake (Hopf 2017; Hopf and Lesscher 2014). On ARD days, animals were given access to drink alcohol containing 10 or 60 mg/L quinine (Bell et al. 2016; Hopf et al. 2010; Radke et al. 2020), randomized across days. These methods are as previously published (Darevsky et al. 2019; Darevsky and Hopf 2020; De Oliveira Sergio et al. 2021; Hopf et al. 2010; Seif et al. 2013; Seif et al. 2015). The 10 mg/L dose of quinine was considered a moderate-challenge and 60 mg/L dose of quinine a higher-challenge condition (Darevsky et al. 2019; Darevsky and Hopf 2020; De Oliveira Sergio et al. 2021; Hopf et al. 2010; Seif et al. 2013).

### Withdrawal

**Figure 2:**
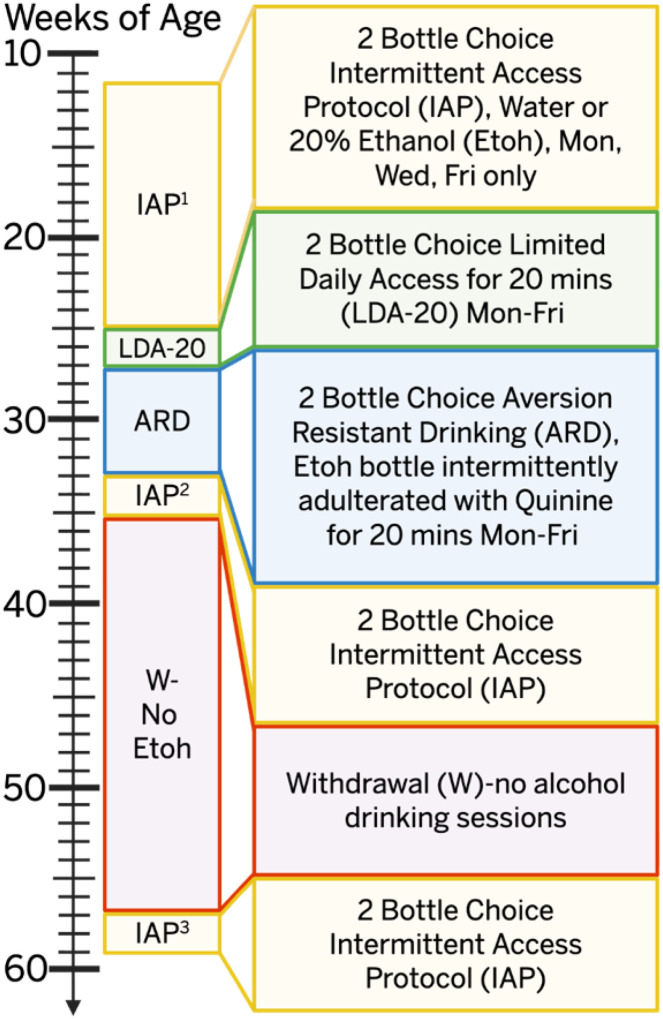
Timeline for animal models of alcohol abuse for all treatment groups.

Following ARD, rats were returned to IAP for a couple of weeks to re-establish stable alcohol drinking (IAP^2^; Fig. 2). Rats then underwent an 18-week withdrawal period where no alcohol-conditioning sessions were administered. Afterwards, rats were returned to IAP for a few weeks (IAP^3^; Fig. 2). Only the rats in ASIR-Early, CON-Early, and ASI underwent withdrawal and subsequent IAP testing. These groups were chosen because ASIR-Early and ASI displayed consistent increases in alcohol intake. Tail blood samples (~ 50 μl) were collected after a 20 min session (not shown in Fig. 2) after completing IAP^3^ to determine blood ethanol concentration (BEC).

### Data Analysis

Each week’s alcohol drinking data for IAP, LDA-20, and ARD were averaged across the days when the 2 bottles were presented. We analyzed data over time using repeated measures (RM) ANOVAs. The factors were *housing* and *week*. We planned comparisons of each treatment group with that group’s initial drinking level (average of first week) because we expected drinking to change over weeks of access. We planned multiple comparisons of each treatment group with its corresponding control *a priori*. Repeated measures ANOVA cannot handle missing values. Our missing values were random in that they occurred when a rat’s data was lost or incomplete due to extraneous logistical circumstances (e.g. bottle spill). We analyzed data sets with missing values by fitting a mixed model as implemented in GraphPad Prism 8.0. This mixed model uses a compound symmetry covariance matrix and was fit using Restricted Maximum Likelihood (REML). In the absence of missing values, this method gave the same P values and multiple comparisons tests as repeated measures ANOVA. In the presence of missing values (missing completely at random), the results can be interpreted like repeated measures ANOVA.

For bar graphs, we analyzed the average of all weeks unless otherwise specified to determine if housing conditions affected total alcohol consumption. Ordinary one-way ANOVAs were used for most analyses. Multiple doses of quinine during ARD were analyzed with a two-way ANOVA. Pre vs Post alcohol withdrawal data were analyzed with a mixed effects model. Blood ethanol content (BEC; mg/dL) was confirmed to be correlated with the alcohol intake (g/kg) as measured by how much fluid was missing from bottles using HPLC-ED following 20 minutes of alcohol drinking and a simple linear regression analysis (r^2^ = 0.7581, p < 0.0001, not shown). The average BEC was approximately 96 mg/dl among the higher ethanol consuming rats (> 1.0 g/kg EtOH). Schematics were made with Biorender.com. Data were plotted as mean ± standard error of the mean (SEM) and analyzed with GraphPad Prism 8.0. Alpha was set at 0.05.

## Results

### ASIR-Early and ASI increase alcohol consumption and preference during IAP^1^

**Figure 3:**
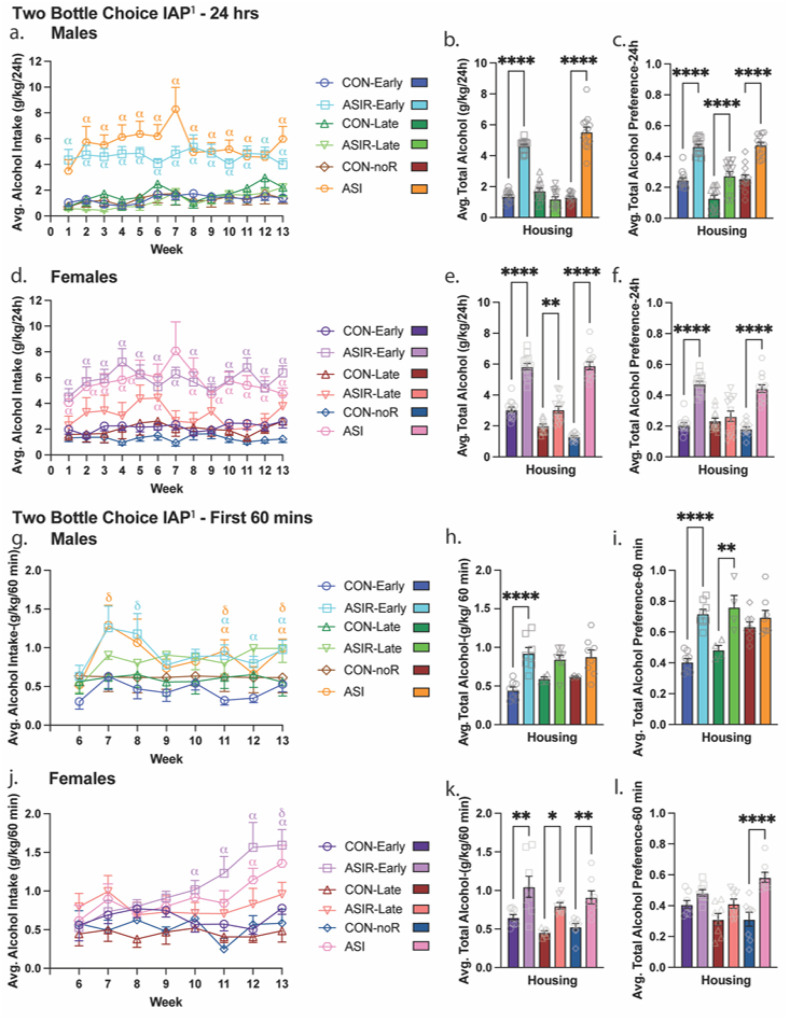
For a through f: 24 hr two bottle choice IAP^1^. Average alcohol intake (g/kg) for three 24 hr sessions per week for males (a) and females (d) a, *p* < 0.05 versus control group for the corresponding housing condition (e.g., ASIR-Early vs. CON-Early), d, *p* < 0.05 versus week 6 within that treatment group, Tukey’s multiple comparisons tests. Average total alcohol intake across all weeks for males (b) and females (e) and average total alcohol preference across all weeks for males (c) and females (f) * *p* < 0.05, **, *p* < 0.01, and ****, *p* < 0.0001 versus group housing condition within the same re-socialization condition, Sidak’s multiple comparisons tests. For g through l: first 60 mins of two bottle choice IAP^1^ test starting on week 6 with statistical details matching corresponding graphs in a through f. N = 12/group. Data are presented as means ± S.E.M.

A primary goal was to compare whether earlier social isolation and re-socialization (P21-P42, ASIR-Early) versus later adolescent social isolation and re-socialization (ASIR-late) differed in their impact on drinking and compared to social isolation throughout adolescent development (ASI) and important controls. ASIR-Early and ASI consistently increased alcohol consumption and preference during the 24 hr two bottle choice intermittent access protocol (IAP^1^) compared to contemporary controls (Fig. 3a–f). Repeated measures (RM) ANOVA revealed a significant main effect of housing for males (F_(5,66)_ = 15.80; *p* < 0.001) with a Tukey’s multiple comparison test indicating significant differences (Fig. 3a) for every week’s average g/kg/24 hr alcohol for ASIR-Early vs Con-Early (*p* < 0.05) as well as almost all weeks for ASI vs. Con-noR (*p* < 0.05). For females, there was also a main effect of housing (RM ANOVA: F_(5,66)_ = 17.56; *p* < 0.001; Fig. 3d), with ASIR-Early different from Con-Early every week (Tukey’s: *p* < 0.05) and ASI was different from Con-noR during all but 1 week (Tukey’s: P < 0.05).

One-way ANOVA was used to compare the average g/kg of alcohol consumed across all weeks of IAP^1^ for males (F_(5,72)_ = 118.8; *p* < 0.0001; Fig. 3b) and females (F_(5,72)_ = 110.2; *p* = 0.0001; Fig. 3e). Sidak’s multiple comparison test indicated that ASIR-Early was different than Con-Early (*p* < 0.0001) and ASI vs Con-noR (*p* < 0.0001) for males (Fig. 3b). For females, ASIR-Early was also different than Con-Early (*p* < 0.0001) and ASI vs Con-noR (P < 0.0001), while ASIR-Late was also different from Con-Late (*p* < 0.01;Fig. 3e). For average alcohol preference, there was a significant effect for males (F_(5,72)_ = 45.67; *p* = 0.0001; Fig. 3c) and females (F_(5,72)_ = 35.36; *p* = 0.0001; Fig. 3f) with all treatment groups being different from their respective controls for males (Tukey’s, *p* < 0.0001), but only ASIR-Early (Tukey’s, *p* < 0.0001) and ASI (*p* < 0.0001) were different from their controls for females (Fig. 3f). Overall, ASIR-Early and ASI consistently increased alcohol consumption and preference during IAP^1^ in both sexes, with only limited effects of ASIR-Late (Figs. 3a–f).

To model front-loading of alcohol consumption, we began measuring the amount of alcohol consumed in the first 60 mins of IAP^1^ session, starting week six (Fig. 3g–l). Weekly averages of alcohol drinking were analyzed with RM ANOVA. There was a significant effect of housing (F_(5,66)_ = 2.84; *p* < 0.05) and time (F_(2.3,124.6)_ = 4.85; *p* < 0.01) for males (Fig. 3g), and also for females (Fig. 3j) for housing (F_(5,66)_ = 6.11; *p* < 0.0001) and time (F_(4.5,244.3)_ = 2.46; *p* < 0.05).

When examining pairwise comparisons overall, males showed greater front-loading at weeks 7 and 8 and at later weeks, while females also showed significant front-loading but only by week 11, and these differences were primarily for ASIR-Early and ASI but not ASIR-Late. Pairwise comparisons for males (Fig. 3g) suggested that ASIR-Early was greater than Con-Early on weeks 11–13 (Sidak’s, *p* < 0.0.05) and ASI was greater than Con-noR on week 11 and 13 (Sidak’s, *p* = 0.003). In addition, ASIR-Early week 8 was different than the first week (week 6; Sidak’s, *p* < 0.05) and ASI weeks 7, 11, and 13 were different than the first week (week 6; Sidak’s, *p* ≤ 0.01). For females (Fig. 3j), ASIR-Early was greater than Con-Early on weeks 10–13 (Sidak’s, *p* < 0.0.05) and ASI was greater than Con-noR on week 12 (Sidak’s, *p* = 0.003). In addition, ASIR-Early week 13 was different than the first week (week 6; Sidak’s, *p* = 0.005). When averaged across all weeks, total 60 minute intake for males (F_(5,34)_ = 9.19; *p* < 0.0001; Fig. 3h) was greater in the ASIR-Early group only compared to their respective control group (Tukey’s, *p* < 0.0001). For females (F_(5,41)_ = 9.35; *p* < 0.0001; Fig. 3k), all groups were greater than their controls (Tukey’s, *p* < 0.05). Total alcohol preference for males (F_(5,34)_ = 13.72; *p* < 0.0001; Fig. 3i) was increased for ASIR-Early and ASIR-Late compared to their controls (Tukey’s, *p* < 0.01). Only the female ASI group was greater than its control (Tukey’s, *p* < 0.0001) for preference (F_(5,42)_ = 9.94; *p* < 0.0001; Fig. 3l). Overall, only the male ASIR-Early and female ASI groups showed both increased alcohol drinking g/kg and preference during the first 60 mins of IAP^1^ sessions (Fig. 3h, i, k & l). Increased alcohol drinking compared to the 6th week for ASIR-Early females and males and ASI males was also observed (Fig. 3g & j).

### Sex dependent effects of ASIR on consumption of alcohol adulterated with quinine

**Figure 4:**
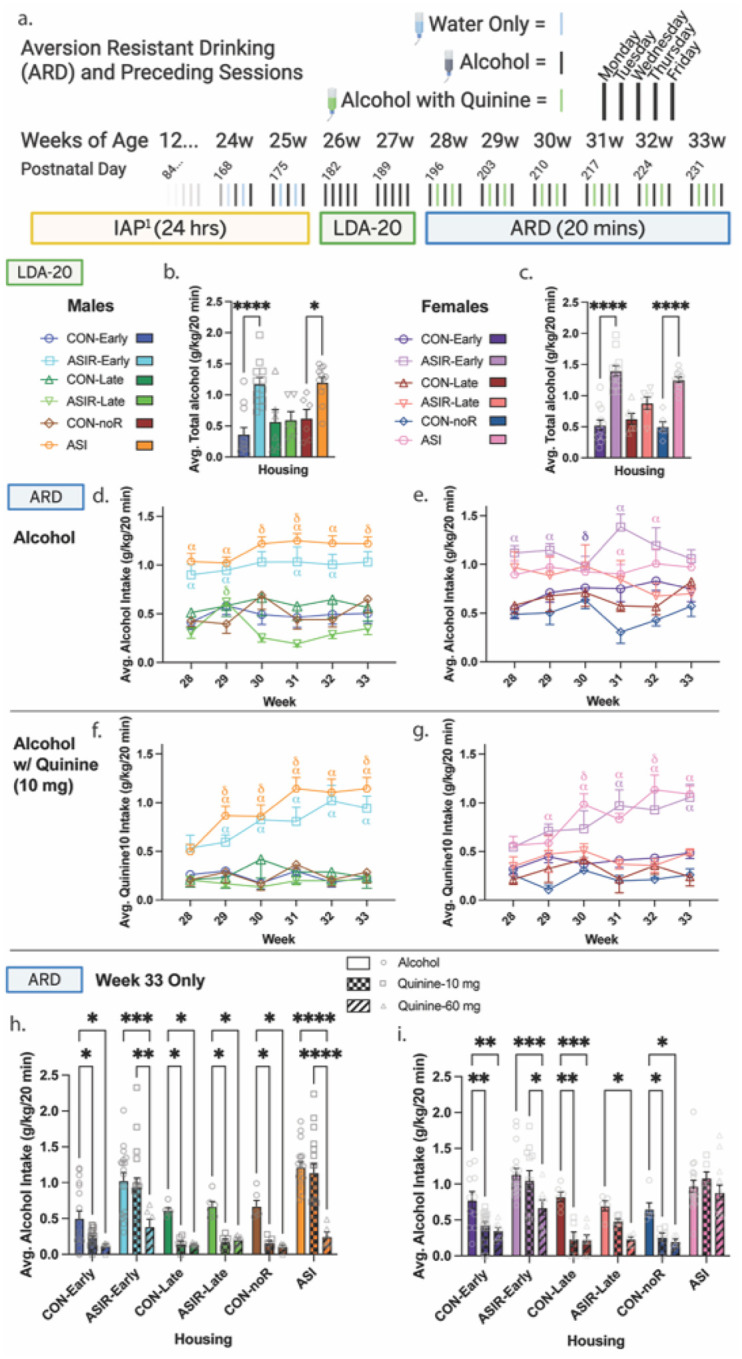
Average alcohol intake during 2 weeks of LDA-20 min for males (a) and females (b). * *p* < 0.05, **, *p* < 0.01, ***, *p* < 0.001, and ****, *p* < 0.0001 versus group housing condition within the same re-socialization condition, Sidak’s multiple comparisons tests (b, c, h & i). Average alcohol consumed per week during 20 min ARD sessions (d & e) and averaged alcohol adulterated with 10 mg/L quinine per week (f & g). alpha, *p* < 0.05 versus control group for the corresponding housing condition, delta, *p* < 0.05 versus week 28 within that treatment group, Tukey’s multiple comparisons tests. Week 33 average alcohol only, 10 mg, and 60 mg quinine adulterated alcohol consumption for males (h) and females (i). Symbols as above, but with Tukey’s. N = 12/group. Data are presented as means ± S.E.M.

We next examined how different housing conditions altered 20-min/day limited access drinking (LDA) and then aversion-resistant drinking (ARD, alcohol with 10 mg/L or 60 mg/L quinine), with ARD tested twice per week (see Methods) to prevent extinction in the lower drinking rats (Fig. 4a). Significant one-way ANOVAs for male (F_(5,48)_ = 10.52; *p* < 0.0001; Fig. 4b) and female (F_(5,48)_ = 27.01; *p* < 0.0001; Fig. 4c) LDA-20 sessions prior to ARD were followed up by Tukey’s multiple comparisons. For LDA-20 in the two weeks before ARD, both ASIR-Early and ASI groups consumed more alcohol than their controls for both males (Tukey’s, *p* < 0.05) and females (Tukey’s, *p* < 0.0001).

During the alcohol-only LDA-20 sessions that were interspersed among ARD sessions (MWF), there was a significant effect of time (F_(3.7,245.8)_ = 3.04; *p* < 0.05), housing (F_(5,66)_ = 11.87; *p* < 0.0001) and an interaction (F_(18.62,245.8)_ = 2.11; *p* < 0.01) for males (Fig. 4d), and a significant effect of housing for females (F_(5,66)_ = 6.20; *p* < 0.0001; Fig. 4e). For males, these groups continued to show elevated LDA-20 alcohol-only intake, compared to week 28, across all other weeks of ARD testing (Fig. 4d) as indicated by a significant RM ANOVA effects of week (F_(3.72,245.8)_ = 3.04; *p* < 0.01), housing (F_(5,66)_ = 11.87; *p* < 0.0001) and an interaction (F_(18.62,245.8)_ = 2.11; *p* < 0.01). ASIR-Early was greater than its control group for all weeks except week 30 (Tukey’s, *p* < 0.05) and ASI was greater than its control for all weeks (Tukey’s, *p* ≤ 0.003) except 30 and 33, for which there were trending toward significance (Tukey’s, *p* = 0.085 and *p* = 0.059, respectively). Male ASI was increased on week 30, 31, and 33 compared to week 28 (Tukey’s, *p* < 0.05), and ASIR-Late week 29 greater than 28 (Tukey’s, *p* < 0.05). For females (Fig. 4e), there was a RM ANOVA effect of housing (F_(5,66)_ = 6.20; *p* < 0.0001) and a trend for an interaction (F_(20.51,230.5)_ = 1.53; *p* = 0.07), with ASIR-Early different from their controls on week 28, 29, and 31 (Tukey’s, *p* ≤ 0.02). Female ASI was different on weeks 31 and 32 (Tukey’s, *p* ≤ 0.03) and Con-Early week 30 was different than week 28 (Tukey’s, *p* = 0.03).

We then examined how housing impacted ARD across weeks of testing. Overall, ARD intake was lower in week one, but increased across weeks, perhaps reflecting a decrease in novelty or aversion of the alcohol-quinine. In addition, consumption of alcohol that was adulterated with quinine (10 mg/L) increased for the ASIR-Early and ASI groups, but no other housing groups, again suggesting a more selective impact of ASIR-Early in particular on increased compulsive-like intake, along with higher alcohol consumption overall. For males (Fig. 4f), there were significant effects of week (F_(3.15, 185)_ = 4.18; *p* < 0.01), housing (F_(5,66)_ = 17.16; *p* < 0.0001) and an interaction (F_(15.73,185)_ = 2.94; *p* < 0.001). ASIR-Early and ASI males were greater than their controls from week 29–33 (Tukey’s, *p* ≤ 0.04). For ASI males all weeks except 32 were significantly greater than the first week (Tukey’s, *p* ≤ 0.01). There were significant effects of week (F_(4.26, 224.2)_ = 3.30; *p* < 0.01) and housing (F_(5,66)_ = 18.35; *p* < 0.0001) for females (Fig. 4g). ASIR-Early females were greater than their controls on week 31 and 33 (Tukey’s, P ≤ 0.05) and ASI females on weeks 29 through 33 (Tukey’s, *p* ≤ 0.01). The ASI females increased on weeks 30 and 32 compared to the first week (Tukey’s, *p* ≤ 0.007). Generally, ASI males and females, and to a slightly lesser extent the ASIR-Early groups, exhibited aversion-resistant drinking and reached a stable level of intake of lower dose quinine-adulterated alcohol by the 6th week.

After 10 mg/L quinine-alcohol drinking plateaued, we also administered higher quinine (60 mg/L) to see how a stronger aversion would impact alcohol consumption in the aversion resistant groups (ASI and ASIR-Early). Overall, 60mg/L quinine substantially reduced alcohol drinking in all male groups, while female ASI, and to a lesser extent ASIR-Early, maintained alcohol drinking despite higher quinine. For males, 2-way ANOVA indicated a significant effect of quinine dose (F_(2, 162)_ = 33.76; *p* < 0.0001), housing (F_(5, 162)_ = 18.16; *p* < 0.0001) and an interaction (F_(10,162)_ = 2.65; *p* < 0.01) (Fig. 4h). Both doses of quinine reduced alcohol drinking in CON-Early, CON-Late, ASIR-Late, and CON-noR (Tukey’s, *p* < 0.05). The ASIR-Early and ASI groups exhibited ARD as the low doses of quinine did not reduce alcohol drinking. Only the high dose reduced drinking in ASIR-Early and ASI (Tukey’s, *p* ≤ 0.0002), which was also significantly lower than the low dose (Tukey’s, *p* ≤ 0.002). For females (Fig. 4i), there was a significant effect of quinine dose (F_(5, 149)_ = 25.25; *p* < 0.0001), housing (F_(5, 149)_ = 23.95; *p* < 0.0001) and an interaction (F_(10, 149)_ = 2.16; *p* < 0.05). Both doses of quinine reduced alcohol drinking in CON-Early, CON-Late, and CON-noR (Tukey’s, *p* < 0.05). While ASIR-Late rats lowered drinking at the low dose of quinine, it did not reach statistical significance until the high dose was administered (Tukey’s, *p* < 0.05). ASIR-Early exhibited ARD as only the high dose reduced drinking (Tukey’s, *p* < 0.001) and was less than the low dose (Tukey's, *p* < 0.05). Female ASI rats did not reduce drinking at any concentration of quinine indicating the most robust ARD behavior (Fig. 4i).

### ASIR-Early and ASI increased alcohol consumption and preference post withdrawal

**Figure 5:**
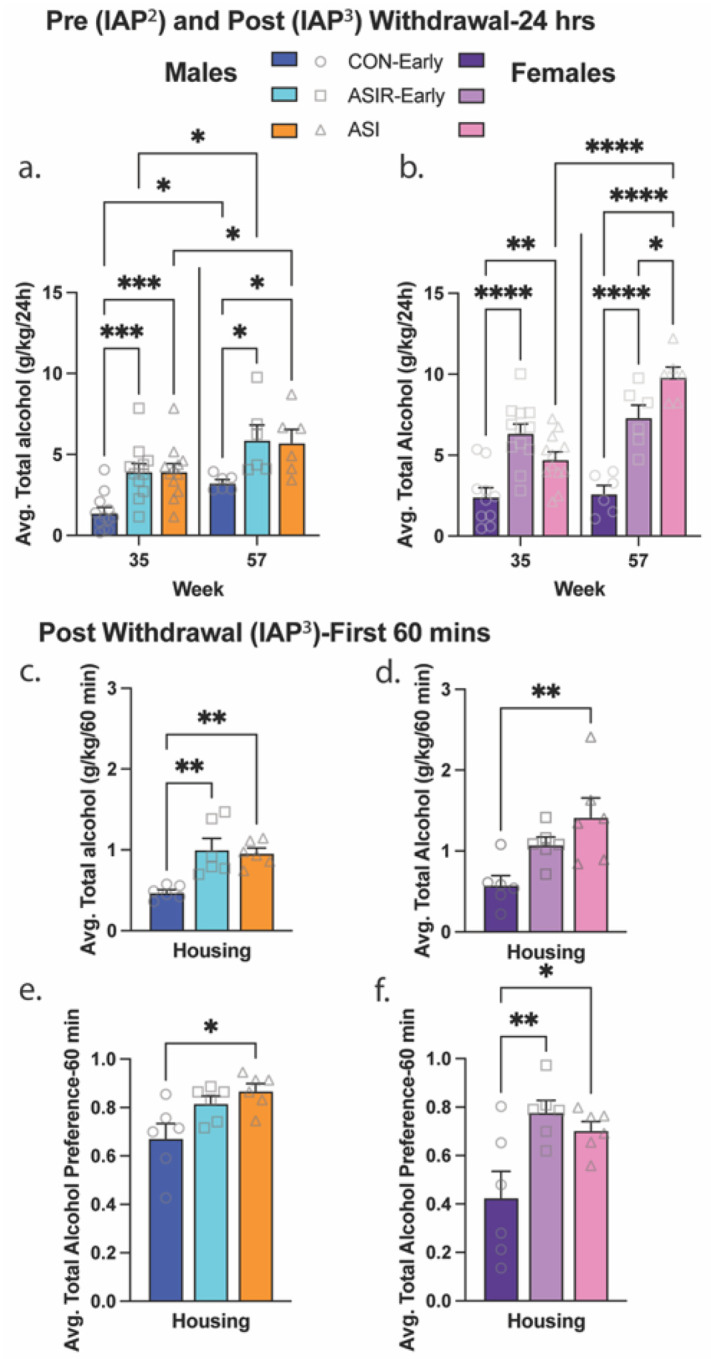
IAP^2^ (week 35) and IAP^3^ (week 57) average weekly alcohol intake for males (a) and females (b). * *p* < 0.05, **, *p* < 0.01, ***, *p* < 0.001, and ****, *p* < 0.0001 versus group housing condition within the same re-socialization condition, Tukey’s multiple comparisons tests. Alcohol consumption during the first 60 mins of the week of IAP^3^ sessions after withdrawal (week 57) for males (c) and females (d). Preference for alcohol over water during the first 60 mins of the first session after withdrawal for males (e) and females (f). Symbols as above, but with Sidak’s multiple comparisons tests. N = 12/group. Data are presented as means ± S.E.M.

Finally, we analyzed alcohol drinking levels during the last week of IAP^2^ (week 35) and compared it to the first week of IAP^3^ (week 57) to determine whether alcohol drinking increased after a long period of no alcohol access, but only in three treatment groups of most interest. Overall, males in all groups tested showed higher 24-hour intake after the 18 weeks abstinence, while interestingly for females only ASI showed higher 24-hour drinking after the long withdrawal. For males (Fig. 5a), there was a significant RM ANOVA effect of week (F_(1, 15)_ = 17.10; *p* < 0.0001) and housing (F_(2, 33)_ = 13.64; *p* < 0.0001). Tukey’s multiple comparisons indicated that on week 35 and 57 ASIR-Early and ASI were greater than CON-Early (P < 0.05) and all three treatment groups increased drinking after the period of withdrawal/abstinence (Tukey’s, *p* < 0.05). For females (Fig. 5b), there was a significant RM ANOVA effect of week (F_(1, 15)_ = 18.04; *p* < 0.001) and housing (F_(2, 31)_ = 36.81; *p* < 0.0001) and an interaction (F_(2, 15)_ = 9.682; *p* < 0.01). Tukey’s multiple comparisons indicated that on week 35 and 57 ASIR-Early and ASI were greater than CON-Early (*p* < 0.01) and ASI was greater than ASIR-Early (*p* < 0.05). Most important, only female ASI rats increased drinking after the period of withdrawal/abstinence (Tukey’s, *p* < 0.0001). Thus, ASIR-CON, ASIR-Early and ASI males escalated alcohol drinking after 22 weeks of withdrawal (Fig. 5a), whereas only the ASI females exhibited this effect (Fig. 5b).

We then examined average alcohol drinking during the first 60 mins of the 24-hour IAP^3^ sessions on week 57 to determine whether frontloading was greater after extended withdrawal/abstinence from alcohol. There was a significant one-way ANOVA effect for g/kg/60min for males (F_(2, 15)_ = 10.90; *p* < 0.01). Tukey’s multiple comparisons indicated that ASIR-Early and ASI were greater than CON-Early (*p* < 0.01; Fig. 5c). There was also a significant effect for females on this same measure (F_(2, 15)_ = 6.98; *p* < 0.01), but only the ASI female rats were significantly greater than CON-Early (Tukey’s, *p* < 0.01). For average total alcohol preference during the same week, there was a significant 1-way ANOVA for males (F_(2, 15)_ = 5.73; *p* < 0.05), but only the ASI group was greater than CON-Early (Tukey’s, *p* < 0.05; Fig. 5e). There was a trend for ASIR-Early vs CON-Early (Tukey’s, *p* = 0.07). For females, there was also a significant main effect (F_(2, 15)_ = 6.78; *p* < 0.01) and both ASIR-Early and ASI were greater than CON-Early (Tukey’s, *p* < 0.01; Fig. 5f). Overall, during the first 60 mins post withdrawal, both ASIR-Early and ASI rats show greater consumption following withdrawal when examining g/kg for males (Fig. 5c) and preference for females in (Fig. 5f).

## Discussion

The present study demonstrates that social isolation during early adolescence produces long-lasting increases in alcohol-related behaviors in adulthood, including escalated alcohol intake, increased alcohol preference, frontloading, and aversion-resistant drinking. In particular, the ASIR-Early and ASI groups showed elevated drinking, while the ASIR-Late largely did not, or did so to a lesser degree. Taken together, these results suggest that isolation during early but not later in the adolescent period is related to risk of higher intake and compulsion. In addition, ASI largely showed the same pattern as ASIR-Early, again suggesting that any condition involving early adolescent isolation increases propensity for alcohol. However, compulsion with higher aversion, and greater drinking after a prolonged abstinence, was only evident in females in the ASI group. This suggests that sustained isolation starting in early adolescence puts females at particular risk of developing alcohol problems such as higher compulsion and relapse after abstinence. Thus, our findings provide important new information about the risk periods during adolescence where isolation increases later drinking. These results also agree with clinical findings that women drinkers often exhibit greater alcohol problems than men (Erol and Karpyak 2015; Evans-Polce et al. 2020; Peltier et al. 2019; White et al. 2015; White 2020), and suggest that adverse early life experiences might be an important contributor.

The ASIR-Early and ASI methods consistently increased alcohol consumption and preference during IAP^1^ compared to contemporary controls (Fig. 3a–f). Only male ASIR-Early and female ASI groups showed both increased alcohol drinking (g/kg) and preference during the first 60 mins of IAP^1^ sessions (Fig. 3h, i, k & l). There was increased alcohol drinking compared to the 6th week for ASIR-Early females and males and ASI males (Fig. 3g & j). In the two weeks before ARD, both ASIR-Early and ASI groups of both sexes were consuming more alcohol than controls (Fig. 4b & c) and these groups continued to be elevated during LDA-20 sessions surrounding the ARD sessions (Fig. 4d & e). Consumption of alcohol that was adulterated with quinine (10 mg/L) increased for the ASI males and females compared week 28 (Fig. 4f & g). By the 6th week, consumption of the low dose of quinine during ARD appears to have plateaued. During week 33, only ASIR-early and ASI males show ARD at the low dose quinine, and the high dose significantly reduces alcohol consumption (Fig. 4h). ASIR-Early, ASIR-Late and ASI females all exhibit ARD, with ASI females resistant even at the high dose of quinine (Fig. 4i). ASIR-CON, ASIR-Early and ASI males escalated alcohol drinking after 22 weeks of withdrawal (Fig. 5a), whereas only the ASI females exhibited this effect (Fig. 5b).

During the first 60 mins of the IAP^3^ sessions, both ASIR-Early and ASI rats show greater consumption following withdrawal when examining g/kg for males (Fig. 5c) and preference for females in (Fig. 5f). Overall, the period during which isolation housing of rats occurs greatly impacts subsequent alcohol drinking. Under these conditions, isolation housing that encompasses the early adolescent period most consistently escalated alcohol intake. For females, ASI increased alcohol drinking the most across all assays (see Figs. 4i and 5b). Whereas for males, ASIR-Early produced the most robust escalation of alcohol drinking (e.g., Fig. 3h & i).

These rats were exposed to IAP to develop a drinking style that models the compulsive alcohol drinking associated with severe alcohol abuse and alcoholism in humans (De Oliveira Sergio et al. 2023; Wise 1973). This model, when applied to Wistar rats for at least 12 weeks, leads to the development of compulsive-like alcohol drinking, whereas continuous alcohol access intake for same amount of time is associated with greater quinine-sensitivity (Hopf et al. 2010). While ASI, in general, typically increases IAP alcohol drinking in rats (Butler et al. 2014a; Lesscher et al. 2015; McElroy et al. 2023; Skelly et al. 2015), it can increase (McCool and Chappell 2009; Ortelli et al. 2023), decrease (Pisu et al. 2011), and have no effect (Gill et al. 2014) using other methods used to establish the alcohol drinking behavior, including increasing alcohol concentrations gradually and adding sweeteners. Thus, when ASI failed to increase IAP drinking (Chandler et al. 2022) it could be because the initial IAP procedure added sweetener to the alcohol bottle. An excellent study in female rats found no increase in IAP drinking, perhaps because the females were not housed singly until P31 (Butler et al. 2014b), in alignment with a lack of elevated drinking in ASIR-Late observed here. Greater IAP drinking was observed in female ASI rats when isolation started early (McElroy et al. 2023). Perhaps a critical period of vulnerability is missed when rats are not isolated early enough in adolescence, which leads little to no escalated alcohol consumption occurring (e.g., current ASIR-Late results and Butler et al. 2014b; Moench and Logrip 2021; Pisu et al. 2011). As early social deprivation is required to observe greater alcohol reward later in life (Whitaker et al. 2013), the increased IAP drinking in ASI and ASIR-Early in both sexes observed here may suggest that the early application of social deprivation is essential for facilitating greater alcohol drinking in adulthood following social isolation.

When switched from 24 hours to 20 mins of daily access to alcohol 2-bottle choice (De Oliveira Sergio et al. 2023), ASIR-Early and ASI rats continued to drink more alcohol. This method relies upon the frontloading style of alcohol drinking and ensures the rats are drinking high levels of alcohol prior to adulterating their alcohol with an aversive stimulus. Some aspects of continuing alcohol intake despite negative consequences are considered a translational method to model human compulsion and addiction for alcohol, compared to IAP (De Oliveira Sergio et al. 2023). Other studies have confirmed that rodents strongly avoid quinine in water at quinine doses that do not reduce alcohol drinking (Hopf et al. 2010; Katner et al. 2022; Lei et al. 2016; Radke et al. 2021; Sneddon et al. 2019). In addition, De Oliveira Sergio, et al. (2024) and other recent work (Domi et al. 2021) found that quinine-water intake with 10 mg/L is reduced 70–75% relative to water alone. These findings concur that this quinine dose greatly reduces water intake, suggesting that alcohol drinking rodents can sense and avoid quinine when in water, while the willingness to continue drinking alcohol adulterated with quinine suggests that their responding is aversion-resistant (compulsion-like) for alcohol (Bell et al. 2016; Hopf et al. 2010; Radke et al. 2020; Radke et al. 2021). The effects of ASI on quinine adulterated alcohol consumption were measured previously. Isolated females demonstrated aversion-resistant drinking when challenged with quinine-adulterated alcohol while ASI males did not (Ortelli et al. 2023). This is somewhat similar to present findings, where both sexes exhibited ARD at the low dose of quinine. However, females in the ASI condition were resistant even at the high dose of quinine. In another valuable study, ARD was examined using foot-shock as the aversive stimulus rather than quinine following ASI (McElroy et al. 2023). Complementing our findings, female mice and rats have greater ARD compared to males (Fulenwider et al. 2019; McElroy et al. 2023). It appears that whether it be quinine or foot-shock, female rats with a history of social isolation may be more prone to ARD. The current results expand on these studies and suggest that ASI starting in early adolescence exacerbates ARD, with higher compulsion especially in females.

The relapse of alcohol drinking after a period of sobriety is an important aspect of the severity of alcoholism and AUD. Animal work has long sought to model this part of human addiction and alcoholism by imposing a drug free period following an extended drug taking period. In animal models of relapse, higher drug taking after abstinence is referred to as the “incubation of drug craving” during a period of abstinence (Chow et al. 2025). When given access to the drug again, the animal will reinstate drug seeking behavior vigorously, and this is considered the animal model of relapse. We note that previously stressed, individually housed female rats exhibited decreased relapse-like alcohol self-administration (Logrip and Gainey 2020), whereas a subset of males increased alcohol self-administration in the same paradigm (Logrip et al. 2014; Logrip and Zorrilla 2012), different from findings here. However, our observation that ASI exacerbated alcohol drinking after a period of abstinence agrees with a past study where adult isolation housing increases alcohol drinking following two weeks abstinence following 8 weeks of IAP (Moench and Logrip 2021). Notably, ours is the first study on ASI during multiple adolescent time periods, and our data support that this rat model of social deprivation during adolescence leads to a greater propensity for relapse later in life, as seen in humans (reviewed in Kirsch et al. 2025).

Re-socialization following isolation housing has been found to “rescue” the individual from the negative impact of the isolation housing, whether it be anxiety-like behavior (open field & elevated plus maze behavior), alcohol drinking, or alcohol conditioned place preference (An et al. 2025). These findings contradict our current results where re-socialization at P42 did not block the increased alcohol drinking caused by isolation, and in some cases caused higher levels of alcohol drinking (e.g., ASIR-Early males IAP^1^). One reason for the discrepancy could be that An et al. (2025) gave alcohol access for two bouts of 5 days. In our study, the difference for females only emerged after many weeks of IAP, during IAP^1^, when looking at frontloading. Further, greater alcohol intake with quinine was absent in the first weeks. While there is some evidence that re-socialization can be similar to social buffering of past adversity, the re-socialization following early ASI perhaps fails to “rescue” upon exposure to alcohol for lengthy periods of time when higher levels of alcohol intake can develop.

Alcohol drinking in humans is often characterized by frontloading or high levels of alcohol consumption to quickly reach the desired level of intoxication. Frontloading can indicate high motivation for alcohol and has been measured in rats to model this human behavior (reviewed in De Oliveira Sergio et al. 2023). This is one of the only studies to investigate sex differences in the impact of ASI of any type on frontloading alcohol drinking behavior in rats. After establishing alcohol drinking, we found that ASIR-Early and ASI groups showed both increased alcohol drinking (g/kg) and preference during the first 60 mins of IAP^1^ sessions (Fig. 3h, i, k & l), with females appearing to ramp up frontloading as the weeks of access continued. These results agree that higher intake early in a session is a learned behavior following extended experience with alcohol drinking.

While neural mechanisms are beyond the scope of the current study, we briefly speculate why we think serotonin (5-HT) is involved. Types of ASI that begin in early adolescence have been found to increases anxiety- and depressive-like behaviors as well as alcohol drinking during adulthood in both sexes by dysregulating the serotonergic (5-HT) system (Lukkes et al. 2012b; Lukkes et al. 2009b; McElroy et al. 2025). Males exposed to ASIR-Early show enhanced inhibition of 5-HT neurons by the stress neuropeptide corticotropin-releasing factor (CRF) (Lukkes et al. 2009c) and chemogenetic activation of DRN 5-HT neurons attenuated reward value for both ethanol and sucrose as well as elevated punished responding for ethanol following ASI (McElroy et al. 2025). Females show reduced activation of basolateral amygdala (BLA) parvalbumin interneurons (PV-I) following an anxiogenic challenge (Lukkes et al. 2012a). In addition, PV-I in the medial prefrontal cortex (PFC) are less excitable following ASI in mice (Kawatake-Kuno et al. 2026). Reduced activity of PV-I, which provides feedforward inhibition of principal neurons, increases BLA excitability (Rainnie et al. 2006). BLA is densely innervated by 5-HT inputs that synapse on and excite PV-I (Jiang et al. 2009; Muller et al. 2007; Rainnie 1999), at least in part via 5-HT receptor type 2A (5-HT2A) (Bocchio et al. 2015). Adult stress leads to reduced 5-HT2A receptor density in the BLA associated with increased anxiety-like behavior (Drevets et al. 2000; Lanzenberger et al. 2007; Morrison et al. 2011; Neumeister et al. 2004; Wu et al. 1999). Reduced 5-HT1A receptor binding has been associated with excessive 5-HT release in dorsal raphe projections, whereas decreased 5-HT2A has been associated with BLA hyperactivity, both implicated in stress-related conditions (Jiang et al. 2009). Importantly, blockade of BLA 5-HT2 receptors in males reduced alcohol self-administration and principal neuron excitability (McCool et al. 2014). Taken together with the current results showing that early, but not late, ASI escalates alcohol consumption, both 5-HT in the dorsal raphe and PV-I in the BLA may be altered by ASI to alter serotonergic control of anxiety-like and alcohol drinking behaviors.

In conclusion, the period during which isolation housing of rats occurs greatly impacts subsequent alcohol drinking. Under these conditions, isolation housing that encompasses the early adolescent period most consistently escalated alcohol intake. For females, ASI increased alcohol drinking the most across all assays. Whereas for males, ASIR-Early best escalated alcohol drinking. In both sexes, isolation at a later adolescent period mostly did not result in higher alcohol intake. Thus, there are sex differences in this model. These results inform future studies that will investigate neural mechanisms underlying social isolation-induced escalation of alcohol drinking. By understanding how the brain works in animal models, our research informs others developing preventive strategies and interventions for adolescents who are at a greater risk of developing AUDs.
